# Two Ounces of Nitrate of Potash Taken at Once

**Published:** 1846-12

**Authors:** 


*Two ounces of Nitrate of Potash taken at once.*—Mr. Gillard communicated to the S. W. branch of the Prov. Med. and Surg. Association, an account of a case in which two ounces of nitrate of potash were taken by a man in mistake for epsom salts. Five minutes after taking it a burning pain in the stomach was felt, which was followed by sickness. The mistake was then discovered. A mustard emetic was administered, followed by carbonate of magnesia and opium. Under this treatment the patient speedily recovered.—*Ibid.*

